# Circulating Small Non-coding RNAs as Biomarkers for Recovery After Exhaustive or Repetitive Exercise

**DOI:** 10.3389/fphys.2018.01136

**Published:** 2018-08-22

**Authors:** Kjell E. J. Håkansson, Ove Sollie, Karin H. Simons, Paul H. A. Quax, Jørgen Jensen, A. Yaël Nossent

**Affiliations:** ^1^Department of Vascular Surgery, Leiden University Medical Center, Leiden, Netherlands; ^2^Einthoven Laboratory for Experimental Vascular Medicine, Leiden University Medical Center, Leiden, Netherlands; ^3^Department of Physical Performance, Norwegian School of Sport Sciences, Oslo, Norway; ^4^Ludwig Boltzmann Cluster for Cardiovascular Research, Vienna, Austria

**Keywords:** snoRNA, exercise physiology, biomarker, microRNA, peripheral artery disease

## Abstract

Circulating microRNAs have proven to be reliable biomarkers, due to their high stability, both *in vivo* in the circulation, and *ex vivo* during sample preparation and storage. Small nucleolar RNAs (snoRNAs) are a different type of small non-coding RNAs that can also be reliably measured in plasma, but have only been studied sporadically. In this study, we aimed to identify RNA-biomarkers that can distinguish between different exercise regimes and that entail clues about muscle repair and recovery after prolonged exhaustive endurance exercise. We compared plasma microRNA profiles between two cohorts of elite cyclists, subjected to two different types of exercise regimes, as well as a cohort of patients with peripheral artery disease (PAD) that were scheduled to undergo lower limb amputation, due to critical limb ischemia. In elite athletes, muscle tissue recovers quickly even after exhaustive exercise, whereas in PAD patients, recovery is completely impaired. Furthermore, we measured levels of a specific group of snoRNAs in the plasma of both elite cyclists and PAD patients. Using a multiplex qPCR screening, we detected a total of 179 microRNAs overall, of which, on average, 161 microRNAs were detected per sample. However, only 30 microRNAs were consistently expressed in all samples. Of these, two microRNAs, miR-29a-3p and miR193a-5p, that responded differently two different types of exercise, namely exhaustive exercise and non-exhaustive endurance exercise. Using individual rt/qPCR, we also identified a snoRNA, SNORD114.1, which was significantly upregulated in plasma in response to endurance exercise. Furthermore, two microRNAs, miR-29a-3p and miR-495-3p, were significantly differentially expressed in athletes compared to PAD patients, but only following exercise. We suggest that these two microRNAs could function as markers of impaired muscle repair and recovery. In conclusion, microRNAs miR-29a-3p and miR-193a-5p may help us distinguish between repeated exhaustive and non-exhaustive endurance exercise. MicroRNA miR-29a-3p, as well as miR-495-3p, may further mark impaired muscle recovery in patients with severe critical limb ischemia. Furthermore, we showed for the first time that a circulating snoRNA, SNORD114.1, is regulated in response to exercise and may be used as biomarker.

## Introduction

Regular exercise at moderate intensity has clear benefits for general health, overall fitness, quality of life and importantly, cardiovascular physiology. Exercise at elite level however, is far more demanding of the body and muscles. During high intensity endurance exercise, muscle glycogen is the major energy substrate and metabolic stress develops. Moreover, exercise triggers an increase in cytokine production, attraction of leukocytes, upregulation of pro-angiogenic stimuli and an overall pro-inflammatory state (Ulven et al., 2015; Langleite et al., 2016). In healthy individuals however, during restitution and repletion of glycogen levels, the inflammation is dampened and over time, muscle function improves.

In patients with peripheral artery disease (PAD), poor circulation leads to a chronic ischemia and low levels of nutrients in the affected limb(s). PAD is caused by chronic accumulation of atherosclerotic lesions in the arteries of the limbs, generally of the leg (Abul-Khoudoud, 2006). Besides lifestyle changes, such as cessation of smoking and increased moderate exercise, and management of risk factors, such as cholesterol lowering, there are no therapeutics available that effectively treat PAD. Patients with severe PAD are at risk of developing critical limb ischemia, defined by cramp-like pain even at rest. Unless blood flow is recovered via physiological neovascularization (i.e., arteriogenesis and angiogenesis), or via surgical revascularization (i.e., stenting or bypass surgery), the pro-inflammatory state becomes permanent and instead of recovery, muscle tissue will start to degrade and ultimately become necrotic. In such severe cases, the only remaining treatment option to prevent sepsis and death is amputation of the affected limb (Germani et al., 2009).

Both in exercise physiology and in PAD pathology, researchers have been trying to identify reliable biomarkers to follow muscle repair and recovery. In exercise physiology, such biomarkers could be used to improve training and recovery schedules to optimize competitive performance. Whereas in PAD, such biomarkers may assist vascular surgeons and PAD patients in their joint decision-making before surgical intervention, it being revascularization or even amputation. Although the respective perspectives are very different, potentially, both fields of research could stand to benefit from each other.

In recent years, circulating microRNAs have proven to be highly reliable biomarkers (Mitchell et al., 2008; Hakimzadeh et al., 2015). MicroRNAs are small, non-coding RNA molecules that inhibit the translation of messenger RNAs by binding to their 3′ untranslated regions (Bartel, 2004). Although microRNAs exert their function intracellularly, microRNAs can also be released into the circulation, either passively upon tissue damage, or actively bound to lipid particles such as HDL, or in microvesicles or exosomes. Microvesicles are also released upon exercise (Wilhelm et al., 2016; Whitham et al., 2018). While extracellular RNA is usually rapidly digested by endogenous RNases, the short length of microRNAs (around 20 nucleotides) protects them from most RNase degradation and gives them relative extracellular stability (Mitchell et al., 2008). Furthermore, microRNA are often highly tissue- or pathway-specificity. Within muscle physiology, muscle-specific microRNAs (myomiRs) such as miR-1 miR-133a, miR-133b, and miR-206 have indeed been detected in the circulation after exercise (Horak et al., 2016). Other studies have shown that non-myomiRs, including the hepatic miR-122, are also increased in the circulation during exercise trials (Cui et al., 2015).

Research into other types of non-coding RNAs, including small nucleolar RNA (snoRNA) has increased as well over the past few years, but little is known about their role in exercise and muscle physiology. Similar to microRNAs, snoRNAs are small, highly conserved RNAs. Their canonical function is to stabilize other RNA species, either through 2′*O*-ribose-methylation (Kiss, 2001), or through pseudouridylation. SnoRNAs have been shown to be reliably measurable in plasma, making them promising candidates for biomarkers, much like microRNAs (Liao et al., 2010).

Besides increased extracellular stability *in vivo*, small non-coding RNAs have also proven to be highly stable *ex vivo*, resisting degradation due to poor sample handling, repetitive freeze-thawing and long-term storage. This remarkable stability may give small non-coding RNAs a further advantage as biomarkers (Peiro-Chova et al., 2013).

In this study, we have performed a screening for circulating microRNAs regulated after exhaustive endurance exercise in a cohort of elite cyclists. Regulated microRNAs were then confirmed and further investigated in a second cohort of elite cyclists performing a 60 min time trial at highest possible intensity. Furthermore, plasma microRNAs were compared between an athlete cohort and a cohort of PAD patients hospitalized for critical limb ischemia, scheduled for lower limb amputation. Finally, we measured levels of a specific group of snoRNAs in the plasma of both elite cyclists and PAD patients.

By comparing the effects of muscle exhaustion on circulating non-coding RNAs between these two extreme ends of the spectrum, from highly trained elite-cyclists to patients in the end-stage of PAD, we aimed identify strong and reliable biomarkers for muscle repair and recovery.

## Materials and Methods

**FIGURE 1:**
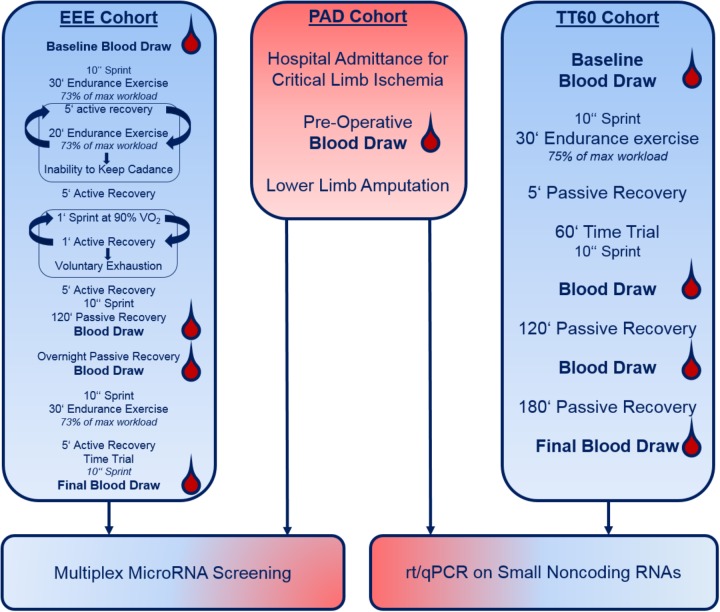
Schematic representation of the study cohorts and set-up. Please note that details on warming-up, etc. are not provided (see section “Materials and Methods”).

A schematic overview of our study set-up is presented in **Figure 1**.

### Exhaustive Endurance Exercise (EEE) Cohort

#### Subjects

Eight male elite endurance cyclists (mean ± SD; age: 22.9 ± 1.2 years; height: 182 ± 2 cm; body mass: 79.5 ± 3 kg) were recruited for our cohort. All subjects had competitive experience at national or international level and trained, 16 ± 1 h weekly, of which 12 ± 2 h was cycling.

This study was carried out in accordance with the recommendations of Guidelines for Human Exercise Trials, Norwegian School of Sport Sciences’ Ethics Committee. The protocol was reviewed by Regional Ethic Committee of Norway (2011/1298) with the decision that the research project was outside the Act on Medical Health Research, confirmed in letter of exemption (2011/1298; IRB Reference No. IRB00006245). The study was conducted according to the Declaration of Helsinki and participants gave their informed written consent after oral and written information about the study.

#### Study Set-Up

Before trial start, subjects completed a familiarization session with a self-paced warm up and a possibility for subjects to adjust the ergometer (Lode Excalibur Sport, Groningen, Netherlands) geometry to individual preferences, as previously described (Rustad et al., 2016). Linear regression was used to identify the workload corresponding to 73% of VO_2max_ (W_73%_).

On the first trial day an exhaustive exercise session was performed. The session started with a warm up as described previously (Rustad et al., 2016). After 5 min of recovery, subjects performed a 10 s all-out effort sprint followed by another 5 min of recovery. The exhaustive exercise session started with 30 min at W_73%_, followed by another 5 min of recovery. Subsequent intervals of 20 min at W_73%_ with 5 min of rest between intervals were performed until subjects were unable to maintain predetermined cadence ± 5RPM. After exhaustion, athletes were given 5 min of recovery, followed by repeated intervals of 60 s at 90% VO_2max_-sprints with 60 s of rest between sprints until voluntary exhaustion. Blood samples were drawn at baseline and at 120 min after the exercise session.

On the second day, roughly 18 h after finishing the first exhaustive exercise session, subjects completed the same warm up as on the previous day. After warm up, 5 min of recovery was followed by a 10 s sprint, followed by 30 min cycling at W_73%_. After 5 min rest, a time test was performed with a predetermined amount of work [Work (kJ) = Power at VO_2max_ (W) ^∗^ 1800 s]. Initial workload was set to W_73%_, but the athletes were free to adjust workload to personal preference. Five minutes after the time-trial participants performed a 10 s all-out effort sprint. Blood was drawn before warm up and immediately after completion of the exercise session.

#### Sample Collection

Blood was drawn from the antecubital vein using a Teflon catheter (18GA, BD Venflon Pro, Franklin Lakes, NJ, United States) into EDTA-coated tubes and centrifuged (2500 *g*, 10 min) at 4°C. Plasma was transferred to 1.5 ml tubes and stored at -80°C until analysis.

### Peripheral Artery Disease (PAD) Cohort – Ampubase

#### Subjects

Sixteen patients (87.5% male; age: 69 ± 13 years) from the Leiden University Medical Center biobank Ampubase were included in our study. All patients were scheduled for lower limb amputation, either below or above the knee joint as a consequence of critical limb ischemia. Inclusion criteria for the biobank were a minimum age of 18 years and lower limb amputation, excluding ankle, foot, or toe amputations. Exclusion criteria were confirmed or suspected malignancy and inability to give informed consent.

The study was approved by the Medical Ethics Committee of the Leiden University Medical Center (Protocol No. P12.265) and written informed consent was obtained from all participants.

#### Sample Collection

Blood samples were collected pre-operatively. Sample collection followed standard hospital procedures. Blood was collected in citrate coated tubes and immediately put on ice. Samples were processed within 30 min of collection and were centrifuged for 10 min at 2800 *g* at 4°C. Plasma was then aliquoted and the plasma closest to the buffy coat was discarded. Aliquots were stored at -80°C until analysis.

### Time Trial (TT60) Cohort

#### Study Population

Thirteen highly trained, male endurance cyclists (mean ± SD; age: 27.7 ± 1.2 years; height: 184 ± 1 cm; body mass: 77.2 ± 1.1 kg) were recruited for this study. All subjects had competitive experience at national or international level and trained for 11.5 ± 1.2 h weekly, of which 8.8 ± 1.4 h was cycling. Due to sample depletion, only 10 athletes with complete sets of samples were included in our analyses.

All subjects were informed of any potential risks before acquiring their written informed consent. The project was approved by Regional Ethics Committee (2013/1528) of Norway, and performed according to the Declaration of Helsinki.

#### Study Set-Up

The initial pre-trial testing, analysis and equipment used in the second cohort was identical to the EEE cohort, described above.

On the day of the trial, subjects started with a standardized warm up of 5–2 min of 50, 55, 60, and 75% of the pre-trial VO_2max_, respectively. The exercise session was started with a 10 s all-out sprint immediately followed by 4 min of active (100W) or passive restitution. After initial restitution, participants cycled 30 min at W_73%_, followed by a 60 min time trial separated by 5 min of restitution. The session ended with another 10 se all-out sprint.

Blood samples were drawn before exercise, immediately after exercise completion as well as after 2 and 5 h of restitution.

#### Sample Collection

Blood was drawn from the antecubital vein using a Teflon catheter (18GA, BD Venflon Pro, Franklin Lakes, NJ, United States) into EDTA-coated tubes. Samples were stored on ice until centrifugation (10 min, 2500 *g*) at 4°C, after which plasma was aliquoted and stored at -80°C until subsequent analysis.

### RNA Isolation and Quantification

Plasma samples from five male EEE Cohort participants and five male Ampubase Cohort participants, were sent to Exiqon Services, Exiqon A/S (Vedbæk, Denmark) for RNA isolation, reverse transcription and Multiplex Circulating MicroRNA qPCR Panel for microRNA expression analysis, using their in-house products and protocols. Samples were selected on relative homogeneity in age and other physical parameters.

Total RNA from 100 μL of plasma was extracted using the miRCURY RNA Isolation Kit – Biofluids (Exiqon, Vedbæk, Denmark), including rDNase treatment to prevent DNA contamination. RNA was stored until further analysis at -80°C.

RNA was reverse transcribed using the miRCURY LNA Universal microRNA PCR, Polyadenylation and cDNA Synthesis Kit. Each microRNA was assayed by qPCR using microRNA Ready-to-Use PCR, Serum/Plasma Focus panel using ExiLENT SYBR Green Master Mix. Negative controls were included as master mix without cDNA template. Amplification was performed using the LightCycler 480 Real-Time PCR System (Roche Molecular Diagnostics, Pleasanton, CA, United States). Amplification curves were analyzed using the Roche LC software, both for determination of Cq and for melt curve analysis.

Amplification efficiency was calculated using Exiqon’s in-house algorithms similar to the LinReg software. Melting curves and temperatures of all assays were within specifications of the assays. All measurements with five Cqs less than the negative control and with a Cq < 37 were included in the analysis. Data that did not meet these criteria were omitted from further analyses. All data were normalized to the average of all assays in all samples (average Cq – assay Cq).

For snoRNA and confirmatory microRNA analyses, total RNA was isolated from all available plasma samples using TRIzol-LS Reagent, according to the Manufacturer’s protocol (Thermo Fisher, Waltham, MA, United States).

For snoRNAs analyses, RNA was reverse transcribed using ‘High-capacity RNA to cDNA kit’ (Thermo Fisher, Waltham, MA, United States) and quantified by RT-qPCR using SybrGreen reagent (Qiagen, Venlo, Netherlands) on the VIIa7 (Thermo Fisher, Waltham, MA, United States). All qPCR reactions were performed in technical triplicates. The Coefficient of Variance (C_v_= Σ/μ) was calculated and triplicates resulting in a C_v_> 0.02 were excluded. SnoRNA expression was normalized to U6.

For confirmatory microRNA quantification, RNA was reversed transcribed and subsequently quantified using microRNA specific Taqman qPCR kits (Thermo Fisher, Waltham, MA, United States). All qPCR reactions were performed in technical triplicates. The C_v_ was calculated and triplicates resulting in a C_v_ > 0.02 were excluded. MicroRNA expression was normalized to miR-191.

### Statistical Analysis

All statistical analyses, unless otherwise specified, were performed using GraphPad Prism 7 (GraphPad Software, Inc., San Diego, CA, United States). *p*-Values of < 0.05 were regarded as significant, *p*-values < 0.1 were regarded as a trend. Normalized relative expression values are presented as mean ± SEM. Due to large inter-sample variation, Grub’s test was used to identify significant (α < 0.05) outliers in ncRNA measurements.

One-way analyses of variance (ANOVA) was used to determine differences in expression levels over time within a cohort. To identify significant effects within ANOVAs, the Tukey’s Honest Significant Difference-test was used as a *post hoc* test for multiple comparisons.

When comparing mean expression between different cohorts two-tailed Student’s *t*-test were used, paired or unpaired, depending on within or between group comparisons. Values are presented as the fold change between groups.

## Results

### Exhaustive Endurance Exercise and Peripheral Arterial Disease

**FIGURE 2:**
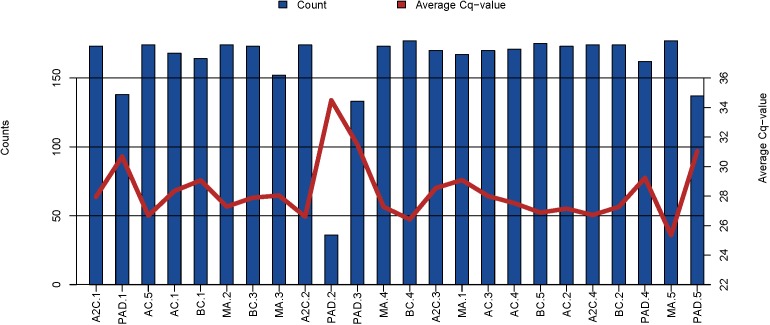
Graphical illustration of the microRNA content in the EEE and PAD cohorts. The blue bars represent the number of microRNAs detected in each sample and the red line shows the average Cq-value for the commonly expressed microRNAs. On average, 161 microRNA were detected per sample. BC, before cycling; AF, after 1^st^ cycling; MA, morning after; A2C, after 2^nd^ cycling; PAD, peripheral artery disease.

Samples drawn from five participants of the EEE and five participants of the PAD cohorts were sent to Exiqon in Denmark for multiplex rt/qPCR expression profiling. The multiplex panel measures a total of 179 microRNA, of which, on average, 161 microRNAs were detected per sample. 30 microRNAs were consistently detected in all samples. Although all samples passed quality control, the microRNA content, both in number of detected microRNAs and expression levels of the detected microRNAs, was lowest in PAD cohort samples (**Figure 2**).

**FIGURE 3:**
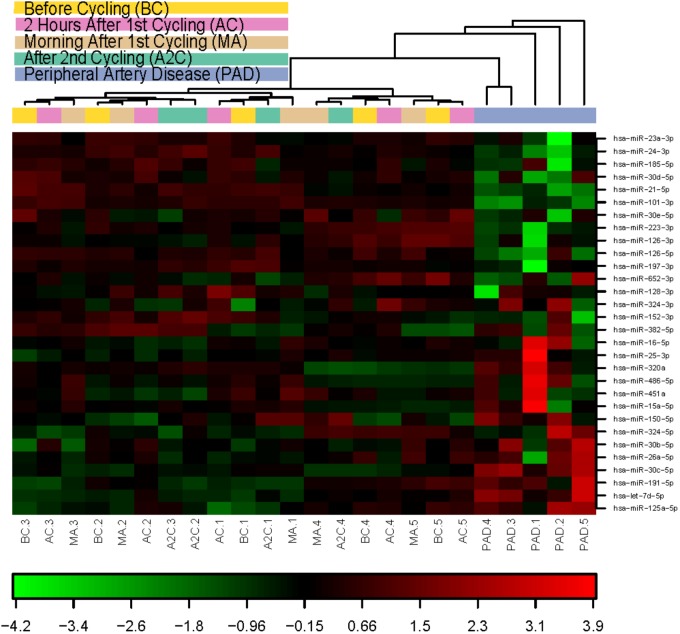
Heat map and unsupervised hierarchical clustering. The clustering was performed on all samples, and on the top 30 microRNAs with highest standard deviation. The normalized expression values (dCq) were used for the analysis. BC, before cycling; AF, after 1^st^ cycling; MA, morning after; A2C, after 2^nd^ cycling; PAD, peripheral artery disease.

When allowing unsupervised clustering on expression data of the 30 microRNAs expressed in all samples, it is obvious from the heatmap (**Figure 3**) that all PAD samples cluster together and are clearly different from the athletes’ samples. When looking at the athletes’ samples, overall clusters are formed, mostly per individual. Inter-individual differences in microRNA profiles are bigger than exercise-induced differences. However, several individual microRNAs were significantly regulated either in response to exhaustive exercise, or in response to recovery (**Tables 1A–C**).

**Table 1:** Regulation of microRNAs in response to exercise.

MicroRNA	Fold change	*p-*Value
**(A) Two hours after compared to before cycling**		
hsa-miR-193a-5p	3.3	0.0066
hsa-miR-29a-3p	1.3	0.0081
**(B) The morning after compared to before cycling**		
hsa-miR-99b-5p	-1.6	0.0074
hsa-miR-151a-3p	-1.2	0.048
**(C) After 2^nd^ cycling compared to before cycling**		
hsa-miR-142-3p	1.3	0.0014
hsa-miR-29a-3p	1.5	0.0026
hsa-miR-106b-5p	-1.3	0.0058
hsa-miR-141-3p	1.6	0.0082
hsa-miR-30d-5p	-1.2	0.011
hsa-miR-150-5p	1.9	0.028
hsa-miR-424-5p	1.6	0.030
hsa-miR-23a-3p	-1.2	0.031
hsa-let-7g-5p	1.3	0.033
hsa-miR-423-3p	1.5	0.034


**Table 1A** shows two microRNAs that are upregulated directly in response to exhaustive exercise, miR-193a-5p (3.3-fold) and miR-29a-3p (1.3-fold). MiR-29a-3p is the only microRNA that is also regulated after the second exercise session (1.5-fold) (**Table 1C**), and thus potentially the most reliable biomarker for muscle exhaustion. As shown in **Table 2C**, multiple microRNAs are regulated in response to a second exercise session. Two microRNAs, miR-99b-5p and miR-151a-3p are downregulated (or depleted) the morning after the first exercise session (-1.6- and -1.2-fold, respectively) (**Table 1B**), indicating that they may be involved in the recovery process. Unfortunately, these microRNAs did not differ, at any time point, from the PAD samples (**Tables 2A–D**), our pathological model for impaired muscle recovery.

**Table 2:** Differential expression of microRNAs between elite cyclists and patients with PAD.

MicroRNA	Fold change	*p-*Value
**(A) Before cycling compared to PAD**		
hsa-miR-483-5p	-21	0.000032
hsa-miR-130a-3p	4.4	0.00031
hsa-miR-2110	-2.5	0.00040
hsa-miR-155-5p	-9.4	0.00060
hsa-miR-19a-3p	5.8	0.00075
hsa-miR-21-5p	3.6	0.0015
hsa-miR-19b-3p	4.6	0.0019
hsa-miR-144-5p	-9.5	0.0020
hsa-miR-148b-3p	2.9	0.0025
hsa-miR-101-3p	4.8	0.0030
hsa-miR-30c-5p	-3.7	0.0035
hsa-miR-590-5p	5.1	0.0037
hsa-let-7a-5p	-4.4	0.0044
hsa-miR-27b-3p	8.6	0.0047
hsa-miR-122-5p	-6.1	0.0059
hsa-miR-193a-5p	-12	0.0064
hsa-miR-335-5p	4.5	0.0082
hsa-miR-106b-5p	2.4	0.0090
hsa-miR-103a-3p	-3.5	0.011
hsa-miR-107	-3.5	0.011
**(B) Two hours after cycling compared to PAD**		
hsa-miR-2110	-2.7	0.000018
hsa-miR-130a-3p	4.5	0.00036
hsa-miR-19a-3p	5.4	0.00063
hsa-miR-144-5p	-12	0.0011
hsa-miR-483-5p	-22	0.0014
hsa-miR-148b-3p	2.9	0.0020
hsa-miR-21-5p	3.3	0.0021
hsa-miR-19b-3p	4.6	0.0022
hsa-miR-101-3p	5.0	0.0023
hsa-miR-122-5p	-5.6	0.0023
hsa-miR-590-5p	5.0	0.0045
hsa-miR-30c-5p	-3.2	0.0052
hsa-miR-27b-3p	8.2	0.0053
hsa-miR-155-5p	-8.5	0.0055
hsa-let-7a-5p	-3.4	0.0060
hsa-miR-335-5p	4.1	0.010
hsa-miR-103a-3p	-3.4	0.011
hsa-miR-106b-5p	2.2	0.012
hsa-miR-107	-3.8	0.012
hsa-miR-495-3p	3.7	0.014
**(C) The morning after compared to PAD**		
hsa-miR-122-5p	-9.8	0.00044
hsa-miR-130a-3p	4.3	0.00065
hsa-miR-483-5p	-23	0.00069
hsa-miR-19a-3p	5.8	0.00082
hsa-miR-155-5p	-5.4	0.0011
hsa-miR-144-5p	-11	0.0017
hsa-miR-21-5p	3.4	0.0019
hsa-miR-101-3p	5.3	0.0019
hsa-miR-30c-5p	-3.9	0.0024
hsa-miR-19b-3p	4.7	0.0028
hsa-miR-145-5p	-2.2	0.0032
hsa-miR-335-5p	6.3	0.0034
hsa-let-7a-5p	-4.4	0.0039
hsa-miR-148b-3p	2.7	0.0039
hsa-miR-27b-3p	7.7	0.0051
hsa-miR-2110	-3.5	0.0059
hsa-miR-590-5p	5.1	0.0069
hsa-miR-885-5p	-11	0.0071
hsa-miR-193a-5p	-11	0.0079
hsa-miR-103a-3p	-3.7	0.0095
**(D) After 2^nd^ cycling compared to PAD**		
hsa-miR-19a-3p	5.9	0.00023
hsa-miR-19b-3p	4.8	0.00068
hsa-miR-122-5p	-8.8	0.0012
hsa-miR-130a-3p	5.6	0.0015
hsa-miR-144-5p	-11	0.0015
hsa-miR-148b-3p	3.0	0.0022
hsa-miR-590-5p	5.8	0.0024
hsa-miR-21-5p	3.6	0.0027
hsa-miR-101-3p	5.2	0.0029
hsa-miR-30c-5p	-3.5	0.0035
hsa-miR-27b-3p	8.2	0.0048
hsa-miR-483-5p	-21	0.0055
hsa-miR-142-5p	6.9	0.0058
hsa-miR-107	-3.7	0.0064
hsa-miR-495-3p	4.5	0.0065
hsa-miR-335-5p	5.3	0.0066
hsa-miR-24-3p	3.3	0.0072
hsa-let-7a-5p	-3.2	0.0075
hsa-miR-2110	-4.1	0.0092
hsa-miR-29b-3p	7.8	0.010


As expected, the number of differentially expressed microRNAs between athletes and PAD patients at any given time point is much larger than between time points within the group of athletes (**Tables 2A–D**). However, there are several changes in circulating microRNA expression profiles. There are several microRNAs that lose their difference to PAD samples directly after the second exercise session. MiR-193a-5p even loses its significant difference from PAD samples already after the first exercise session. One specific microRNA, miR-495-3p is significantly upregulated in response to both exercise sessions, compared to the PAD samples (3.7- and 4.5-fold, respectively).

The microarray data are available via the Gene Expression Omnibus (GEO^1^) via Accession No. GSE117654.

### Time Trial Exercise and Peripheral Arterial Disease

We selected nine microRNAs to be measured in a second, but different exercise cohort, where elite cyclists were subjected to a strenuous, but non-exhaustive, time trial. MiR-29a-3p, miR-193a-5p, miR-495-3p, miR-106b-5p, and miR-155-3p were selected based on our findings in the EEE cohort. MiR-1-3p, miR-133a-3p, miR-133b-3p, and miR-122-5p were selected based on literature describing their regulation in plasma in response to exercise (Cui et al., 2015; Horak et al., 2016).

**FIGURE 4:**
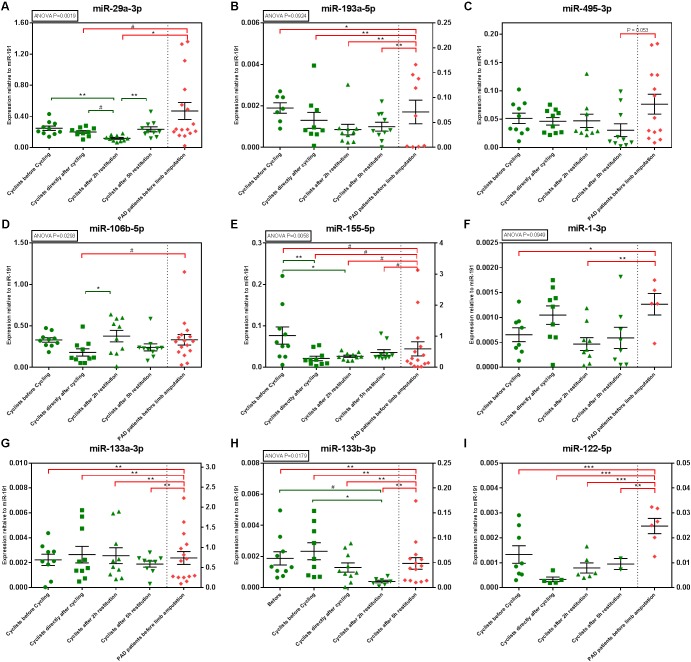
MicroRNA expression in the TT60 and PAD cohorts. Individual sample values are represented by green (athletes) and red (PAD patients) symbols; means with their corresponding SEMs are represented by black lines. ANOVAs were performed to determine circulating microRNA regulation over time and Tukey’s Honest Significant Difference-tests were performed to determine differences between time points, within the TT60 cohort (*N* = 10; but not all samples gave detectable microRNA levels at all time points). Differences between the PAD cohort (*N* = 16; but not all samples gave detectable microRNA levels at all time points) and different time points of the TT60 cohort were analyzed using unpaired, two-tailed Student’s *t*-tests. #*p* < 0.1; ^∗^*p* < 0.05; ^∗∗^*p* < 0.01; ^∗∗∗^*p* < 0.001. **(B,E,G–I)** The TT60 and PAD cohort levels are expressed on separate X-axes.

MiR-29a-3p and miR-193a-5p were both upregulated at 2 h after exhaustive exercise (**Figure 4**; ANOVA *p* = 0.0019 and *p* = 0.0924, respectively). Two hours after the time trial however, both microRNAs were significantly downregulated. Both miR-106b-5p and miR-155-5p were also downregulated after the time trial, but both microRNAs normalized rapidly (**Figure 4**; ANOVA *p* = 0.0293 and *p* = 0.0058, respectively).

Of the ‘myomiRs,’ only miR-133b-3p was regulated significantly in response to the time trial, although a similar trend was observed for miR-1-3p (ANOVA *p* = 0.0179 and *p* = 0.0949, respectively). Both microRNAs appeared upregulated directly after the time trial and decreased in expression during recovery.

When compared to the PAD cohort, we again observed that microRNA expression profiles differ strongly between elite athletes and patients with critical limb ischemia. Most of the microRNAs that we measured, including miR-193a-5p, miR155-5p, miR133a-3p, miR-133b-3p, and miR-122-5p were lower in athletes than in PAD patients at all time points, both before and after exercise. Consistent with the EEE cohort however, miR29a-3p and miR-495-3p only became different from the PAD cohort after exercise. MiR-29a-3p was significantly lower in the TT60 cohort than in the PAD cohort, directly and at 2 h after the time trial. MiR-495-3p was borderline-significantly lower (*p* = 0.053) in the TT60 cohort than in the PAD cohort in the later stages of recovery, at 5 h after the time trial.

### SnoRNA Expression in the TT60 and PAD Cohorts

SnoRNAs are also small non-coding RNA molecules that can be reliably measured in human plasma (Liao et al., 2010). As ‘snoRnome-wide’ screening methods are not yet readily available, we selected a specific subset of snoRNAs and measured these by rt/qPCR, in order to determine whether or not snoRNAs may be suitable as biomarkers for either exercise or muscle recovery. We selected seven snoRNAs that are expressed from a single gene locus, 14q32, which also encodes 54 microRNA genes, including miR-495-3p, that we know are involved in angiogenesis and vascular remodeling (Welten et al., 2014, 2017).

All seven snoRNAs could indeed be detected in human plasma. However, especially in the exercise cohort, some snoRNAs were expressed below detection levels in some individual athletes. One snoRNA, SNORD114.1 was significantly upregulated over time after the time trial. SNORD114.1 levels increased gradually throughout the recovery period (*p* = 0.022).

**FIGURE 5:**
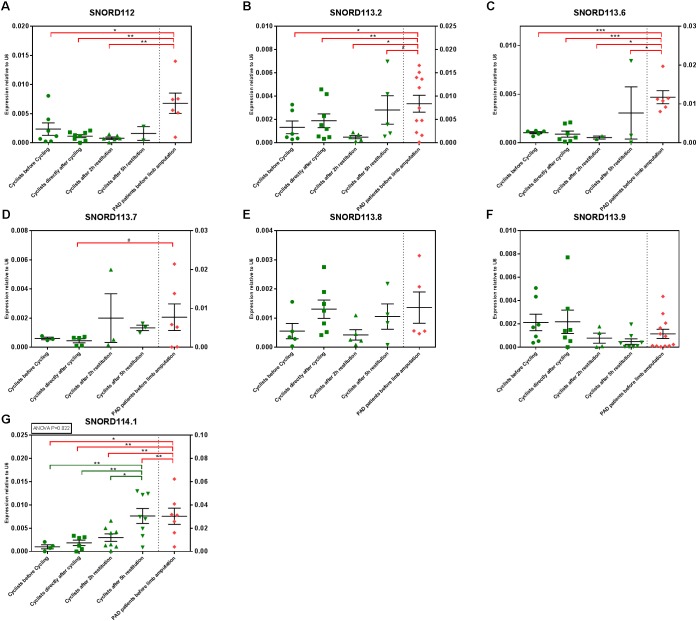
SnoRNA expression in the TT60 and PAD cohorts. Individual sample values are represented by green (athletes) and red (PAD patients) symbols; means with their corresponding SEMs are represented by black lines. ANOVAs were performed to determine circulating snoRNA regulation over time and Tukey’s Honest Significant Difference-tests were performed to determine differences between time points, within the TT60 cohort (*N* = 10; but not all samples gave detectable snoRNA levels at all time points). Differences between the PAD cohort (*N* = 16; but not all samples gave detectable snoRNA levels at all time points) and different time points of the TT60 cohort were analyzed using unpaired, two-tailed Student’s *t*-tests. #*p* < 0.1; ^∗^*p* < 0.05; ^∗∗^*p* < 0.01; ^∗∗∗^*p* < 0.001. **(B–D,G)** The TT60 and PAD cohort levels are expressed on separate X-axes.

When comparing athletes to PAD patients, the snoRNAs were either different between both cohorts at all TT6o time points (SNORD112, SNORD113.2, SNORD113.6, and SNORD 114.1) or at none or the TT60 time points (SNORD113.7, SNORD113.8, and SNORD113.9). Expression levels of the snoRNAs are presented in **Figure 5**.

## Discussion

In this study, we explored the suitability of circulating small non-coding RNAs, including microRNA and snoRNAs, as biomarkers for exercise and muscle damage. We included two cohorts of elite cyclists, subjected to two different types of exercise regimes, as well as a cohort of patients with PAD that were scheduled to undergo lower limb amputation, due to critical limb ischemia. We identified several microRNAs, as well as one snoRNA, that were regulated in plasma in response to strenuous endurance exercise. Furthermore, we identified microRNAs that may indicate impaired suboptimal muscle repair, as they were only differentially regulated from PAD patients, during the recovery phase in elite cyclists.

The first thing we noticed, when looking at the microRNA profiling data after repeated exhaustive endurance exercise, was the fact that differences in microRNA expression profiles are much bigger between individuals than between the different time points before, during, and after the 2-day exercise regime. Although we did identify several microRNAs that were significantly regulated in response to exercise, our group of athletes was quite homogenous, consisting only of male elite cyclists in their early twenties. MicroRNA expression is highly variable between individuals and is often age-dependent. Therefore, future replication studies in larger and more heterogeneous cohorts are necessary to determine the most reliable microRNA-biomarkers.

We did identify two microRNAs that were significantly regulated in response to the first exhaustive exercise session, miR-29a-3p and miR-193a-5p. MiR-29a-3p was the most consistently regulated, as it was also upregulated on the second day. MiR-29a-3p had also previously been shown to be regulated in response to exercise (de Gonzalo-Calvo et al., 2015). Both microRNAs were also regulated in the second exercise cohort, in response to a 60 min time trial. However, after non-exhaustive endurance exercise, both miR-29a-3p and miR-193a-5p were downregulated in plasma. Although surprising, this is a promising finding, because this could mean that levels of miR-29a-3p and miR-193a-5p may help distinguish between different exercise regimes and indicate the extent of muscle exhaustion in athletes.

MiR29a-3p, like the other microRNAs of the miR-29-family, plays a crucial role in the formation and maintenance of the extracellular matrix and controls, amongst others, the production of collagens. Less is known about the function of miR-193a, but a recent study has shown that miR-193a interacts with High mobility group box-1 (HMGB1) and has effects on angio- and vasculogenesis (Khoo et al., 2017). HMGB1 is a well-described nuclear protein with a central role in inflammation and apoptosis (Yang et al., 2013). Whether or not miR-29a-3p and miR-193-5p play a functional role in muscle physiology, or if they are markers for changes in the musculature remains to be determined.

A third microRNA, miR-106b-5p could potentially provide information about the recovery after exercise. MiR-106b-5p is downregulated in response to both exhaustive and endurance exercise, but only immediately after exercise completion (after the second exercise session) and to the 60 min time trial. However, 2 h after the first exhaustive exercise session, and also 2 h after completion of the 60 min time trial, miR-106b-5p have already normalized to the same levels as before exercise. MiR-106b-5p has previously been shown to be highly expressed in skeletal muscle of diabetic patients (Gallagher et al., 2010) and mouse studies show that miR-106b overexpression result in skeletal muscle mitochondrial dysfunction and insulin resistance (Zhang et al., 2013). Exercise has a well-known effect on insulin resistance and sensitivity (Borghouts and Keizer, 2000) and possibly, miR-106-5p plays a role in this process.

When looking at the more classic myomiRs, only miR-133b-3p and possibly miR-1-3p were regulated in response to exercise after the 60 min time trial. But then again, reproducibility in exercise biomarker studies appears relatively low throughout literature. Many different microRNAs, as well as many different cytokines have been reported to associate with various forms of exercise over the years (Ultimo et al., 2018). One explanation could be that different forms and intensities of exercise can have profoundly different physiological effects. Another explanation could be that inter-individual differences are simply larger than differences induced by the exercise regimes. Indeed, in our unsupervised clustering of microRNA expression (**Figure 3**), we observed that microRNAs are clustered by individual, rather than by time point.

A major finding in the present study was that exercise influences plasma snoRNA levels. snoRNAs have previously been described to be stable in the circulation (Liao et al., 2010), however, this is the first report of exercise-mediated regulation of snoRNAs. SnoRNAs may provide more reliable biomarkers in the future. Out of seven snoRNAs, SNORD114.1 is significantly upregulated in response to exercise and increases further during recovery. These seven snoRNAs were selected because they are transcribed from the same gene locus as microRNA miR-495-3p. However, if snoRnome-wide screening methods were to become commercially available, snoRNAs may offer much more information on muscle condition and recovery. However, further exploratory studies are certainly necessary to establish circulating snoRNAs as biomarkers for any form of exercise.

Finally, we identified two microRNAs that may function as markers of impaired muscle repair and recovery. Both miR-29a-3p and miR-495-3p are significantly different in athletes compared to PAD patients, but only following exercise. The fact that these microRNAs differ significantly from PAD patients specifically during the recovery stage indicates that plasma levels of these microRNAs may be a marker for muscle repair. Especially miR-495-3p is a promising candidate to mark impaired muscle repair and end-stage PAD, as we have previously shown that miR-495-3p is highly functional in a mouse model for severe PAD (Welten et al., 2014). Inhibition of this microRNA increases endogenous ischemia-induced neovascularization and significantly increased angiogenesis and muscle perfusion. MiR-495-3p is significantly upregulated in response to both exercise sessions, compared to the PAD samples, which indicates that miR-495-3p could be associated with repair processes that are lacking in patients with severe PAD.

Our study design has several limitations. First, we did not include non-exercise control groups in our exercise cohorts. Of course, we measured levels of microRNAs and snoRNAs in all participants before starting the different exercise regimes, but as samples were taken at different time point over 2 days, we could not correct for potential confounding factors like circadian rhythm. Similarly, due to our study design where only PAD patients scheduled for limb amputation surgery were included in the study, we did not include samples from PAD patients at other time points. Finally, due to sample depletion, we were unable to confirm the findings from the microarray analyses by qPCR in all the samples of the EEE cohort.

## Conclusion

In this study, we have investigated the regulation of circulating small non-coding RNAs in response to exercise. MicroRNAs miR-29a-3p, miR-193a-5p, and miR-106b-5p have shown to be significantly regulated in the circulation in response to endurance exercise. MiR-29a-3p and miR-193a-5p may even be able to distinguish between repeated exhaustive and non-exhaustive exercise. MicroRNAs miR-29a-3p and miR-495-3p may mark impaired muscle recovery in patients with severe critical limb ischemia. Furthermore, we showed for the first time that a circulating snoRNA, SNORD114.1, is regulated in response to endurance exercise.

## Author Contributions

KH performed the experiments, analyzed the data, and wrote the manuscript. OS performed the experiments, collected the samples, and edited the manuscript. KS collected the Ampubase biobank samples and edited the manuscript. PQ supervised the Ampubase biobank samples collection and edited the manuscript. JJ designed the protocols, supervised the experiments, and edited the manuscript. AN designed the protocols, supervised the experiments, and wrote the manuscript.

## Conflict of Interest Statement

The authors declare that the research was conducted in the absence of any commercial or financial relationships that could be construed as a potential conflict of interest.

## Footnotes

1 https://www.ncbi.nlm.nih.gov/geo/
